# Aurora Kinases as Targets in Drug-Resistant Neuroblastoma Cells

**DOI:** 10.1371/journal.pone.0108758

**Published:** 2014-09-30

**Authors:** Martin Michaelis, Florian Selt, Florian Rothweiler, Nadine Löschmann, Benedikt Nüsse, Wilhelm G. Dirks, Richard Zehner, Jindrich Cinatl

**Affiliations:** 1 Institut für Medizinische Virologie, Klinikum der Goethe-Universität, Frankfurt am Main, Germany; 2 Centre for Molecular Processing and School of Biosciences, University of Kent, Canterbury, United Kingdom; 3 Leibniz-Institute Deutsche Sammlung für Mikroorganismen und Zellkulturen GmbH, Braunschweig, Germany; 4 Institut für Rechtsmedizin, Klinikum der Goethe-Universität, Frankfurt am Main, Germany; Institut de Génétique et Développement de Rennes, France

## Abstract

Aurora kinase inhibitors displayed activity in pre-clinical neuroblastoma models. Here, we studied the effects of the pan-aurora kinase inhibitor tozasertib (VX680, MK-0457) and the aurora kinase inhibitor alisertib (MLN8237) that shows some specificity for aurora kinase A over aurora kinase B in a panel of neuroblastoma cell lines with acquired drug resistance. Both compounds displayed anti-neuroblastoma activity in the nanomolar range. The anti-neuroblastoma mechanism included inhibition of aurora kinase signalling as indicated by decreased phosphorylation of the aurora kinase substrate histone H3, cell cycle inhibition in G2/M phase, and induction of apoptosis. The activity of alisertib but not of tozasertib was affected by ABCB1 expression. Aurora kinase inhibitors induced a p53 response and their activity was enhanced in combination with the MDM2 inhibitor and p53 activator nutlin-3 in p53 wild-type cells. In conclusion, aurora kinases are potential drug targets in therapy-refractory neuroblastoma, in particular for the vast majority of p53 wild-type cases.

## Introduction

Since their discovery in 1995, the aurora kinases have gained much interest as drug targets in cancer. In humans, there are three known homologous family members, the aurora kinases A, B, and C. They are involved in the organisation of the spindle apparatus during mitosis. Various aurora kinase inhibitors are under pre-clinical and clinical investigation [1], [2].

Neuroblastoma is the most frequent extracranial solid childhood tumour. About half of patients suffer from high-risk disease associated with overall survival rates below 50% despite intensive therapy [3], [4]. MYCN amplification is a major negative prognostic factor in neuroblastoma indicating high-risk disease [3], [4]. Aurora kinase A expression and amplification were shown to be negative prognostic markers in neuroblastoma and to stabilise MYCN [5], [6]. Moreover, Aurora kinase B was identified as drug target in neuroblastoma tumour-initiating cells with deregulated BRCA1 signalling [7]. Different aurora kinase inhibitors including the aurora kinase A inhibitors MLN8054 and alisertib (MLN8237), the aurora kinase B inhibitor AZD1152, and the pan aurora kinase inhibitor CCT137690 were demonstrated to display anti-neuroblastoma activity [5], [7]–[15].

Resistance acquisition is a major problem in neuroblastoma [3], [4] and aurora kinase inhibitors have not been investigated in neuroblastoma models of acquired resistance. Here we tested tozasertib (VX680, MK-0457), a pan aurora kinase inhibitor [16], and alisertib, a second generation aurora kinase inhibitor that inhibits aurora kinase A and B with a higher affinity to aurora kinase A [17], in a panel of drug-resistant neuroblastoma cell lines.

## Materials and Methods

### Drugs

Tozasertib, alisertib, and nutlin-3 were purchased from Selleck Chemicals (Houston, Tx, USA), cisplatin and vincristine from Gry-Pharma GmbH (Kirchzarten, Germany), and doxorubicin from Cell-Pharm GmbH (Bad Vilbel, Germany).

### Cells

The MYCN-amplified neuroblastoma cell lines UKF-NB-2, UKF-NB-3, and UKF-NB-6 were established from stage 4 neuroblastoma patients [18]–[20]. Parental chemosensitive cell lines were adapted to growth in the presence of anti-cancer drugs by continuous exposure of these cell lines to the increasing concentrations of these drugs as described before [18], [19], [21].

The following drug-adapted neuroblastoma cell lines were derived from the resistant cancer cell line (RCCL) collection (www.kent.ac.uk/stms/cmp/RCCL/RCCLabout.html): UKF-NB-2^r^DOX^20^ (doxorubicin), UKF-NB-2^r^VCR^10^ (vincristine), UKF-NB-3^r^CDDP^1000^ (cisplatin), UKF-NB-3^r^DOX^20^, UKF-NB-3^r^VCR^10^, UKF-NB-6^r^CDDP^2000^, UKF-NB-6^r^VCR^10^ [18], [19], [22], UKF-NB-3^r^Nutlin^10µM^ (nutlin-3), UKF-NB-6^r^Nutlin^10µM^ [21], UKF-NB-2^r^Nutlin^10µM^ (established as described in [21]). ABCB1 expression and p53 status of the cell lines are provided in Table S1.

All cells were propagated in IMDM supplemented with 10% FBS, 100 IU/ml penicillin and 100 mg/ml streptomycin at 37°C. Cells were routinely tested for mycoplasma contamination and authenticated by short tandem repeat profiling.

p53-depleted cells or cells showing high expression of ABCB1 (also known as MDR1 or P-glycoprotein) were established as described previously [21], [23], [24] using the Lentiviral Gene Ontology (LeGO) vector technology [25], [26] (www.lentigo-vectors.de).

### Viability assay

Cell viability was tested by the 3-(4,5-dimethylthiazol-2-yl)-2,5-diphenyltetrazolium bromide (MTT) dye reduction assay after 120 h incubation modified as described previously [21], [22].

### qPCR

Total RNA was isolated from cell cultures using TRI reagent (Sigma-Aldrich, München, Germany). Quantitative real-time reverse transcriptase PCR (qPCR) for viral mRNA was performed as described previously [27] using the following primers: 18 s ribosomal RNA, forward primer 5′ gtg aaa ctg cga atg gct cat 3′, reverse primer 5′ ctg acc ggg ttg gtt ttg at 3′; CDKN1A (p21), forward primer: 5′ gcc cgt gag cga tgg aa 3′, reverse primer 5′ acg ctc cca ggc gaa gtc 3′; BAX, forward primer 5′ agt aac atg gag ctg cag agg at 3′, reverse Primer 5′ gct gcc act cgg aaa aag ac 3′; BBC3 (PUMA), forward primer: 5′ ggg ccc gtg aag agc aa 3′, reverse primer: 5′ gga gca acc ggc aaa cg 3′; GADD45, forward primer: 5′ gca cgc cgc gct ctc t 3′, reverse primer 5′ ctt atc cat cct ttc ggt ctt ctg 3′; MDM2, forward primer 5′ tgt tgg tgc aca aaa aga ca 3′, reverse primer 5′ cac gcc aaa caa aca aat ctc cta 3′; PMAIP1 (NOXA), forward primer 5′ gaa gaa ggc gcg caa gaa 3′, reverse primer 5′ tgc cgg aag ttc agt ttg tct 3′.

### Western blot

Cells were lysed in Triton X-sample buffer and separated by SDS-PAGE. Proteins were detected using specific antibodies directed against β-actin (BioVision via BioCat GmbH, Heidelberg, Germany), p21 (Cell Signaling via New England Biolabs, Frankfurt am Main, Germany), and p53 (Enzo Life Sciences, Lörrach, Germany) and were visualised by enhanced chemiluminescence using a commercially available kit (Amersham, Freiburg, Germany).

### Flow cytometry

The cells were fixed and permeabilised using Cytofix/Cytoperm (BD Biosciences, Heidelberg, Germany) according to the manufacturer's protocol. To detect Bax activation, the cells were incubated with a mouse monoclonal anti-Bax antibody (clone 6A7, BD Pharmingen, Heidelberg, Germany) for 30 min at 4°C that specifically recognises a Bax binding site that is exclusively exposed upon Bax activation in the mitochondrial membrane. To detect histone H3 phosphorylation, the cells were incubated with a mouse monoclonal anti-phosphohistone H3 antibody (Cell Signaling, EMD Millipore Corporation, Billerica, MA, USA) for 30 min at 4°C. After washing and incubation with a secondary phycoerythrin (PE)-labeled anti-mouse antibody for 30 minutes at 4°C, the percentage of cells displaying activated BAX or phosphorylated histone H3 were quantified by flow cytometry.

For cell cycle analysis, cells were fixed with 70% ice-cold ethanol over night and stained with 20 µg/mL propidium iodide (Calbiochem, Merck KgaA, Darmstadt, Germany). The DNA content was determined by flow cytometry.

All experiments were performed using a FACSCanto (BD Biosciences, Franklin Lakes, NJ, USA).

### Caspase 3/7 activity assay

The activity of the caspases 3 and 7 was examined using the Caspase-Glo 3/7 Assay (Promega GmbH, Mannheim, Germany) following the manufacturer's instructions. Cells were seeded in 96-well cell culture plates and allowed to adhere overnight. After drug treatment, the culture plates were adjusted to room temperature. Then, the cells were incubated for 5 min with the pre-mixed substrate and the luminescent signal was measured with a plate reader (Tecan, Crailsheim, Germany) for 30 cycles.

### Statistics

Results are expressed as mean ± S.D. of at least three experiments. Comparisons between two groups were performed using Student's t-test. Three and more groups were compared by ANOVA followed by the Student-Newman-Keuls test. P values lower than 0.05 were considered to be significant.

## Results

### Effects of tozasertib and alisertib on parental neuroblastoma cell lines and their drug-resistant sub-lines

**Figure 1:**
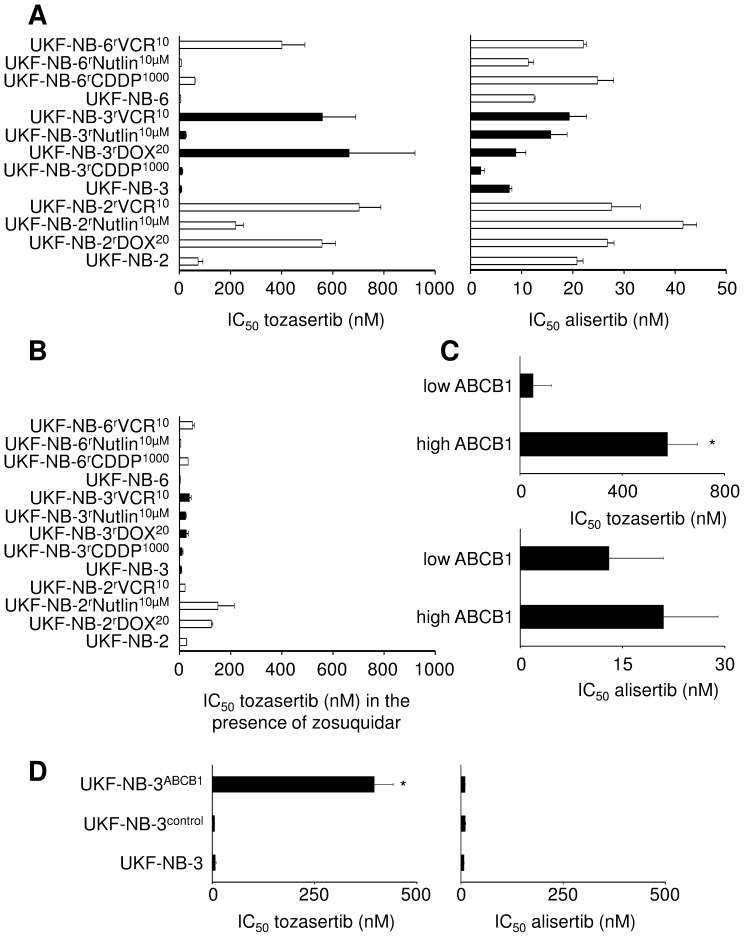
Effects of tozasertib and alisertib on neuroblastoma cell viability. A) IC_50_ values determined after 120 h of incubation by MTT assay; B) tozasertib IC_50_ values in the presence of the ABCB1 inhibitor zosuquidar (5 µM); C) Average IC_50_ values in high and low ABCB1-expressing cells, * P<0.05 compared to low ABCB1-expressing cells; D) IC_50_ values in UKF-NB-3 cells, UKF-NB-3 cells transduced with a lentiviral vector encoding for ABCB1 (UKF-NB-3^ABCB1^), and UKF-NB-3 cells transduced with a control vector (UKF-NB-3^control^), * P<0.05 compared to UKF-NB-3.

The IC_50_ values of tozasertib and alisertib were determined in a panel of neuroblastoma cell lines and their drug-resistant sub-lines (Figure 1A, Table S1). The tozasertib IC_50_ values displayed a much wider distribution (5.5±0.4 nM to 664.0±257.8 nM) than the alisertib IC_50_ values (7.6±0.5 nM to 26.8±1.3 nM). The relative resistance (IC_50_ resistant sub-line/IC_50_ respective parental cell line) ranged for tozasertib from 1.4 to 92.2 and for alisertib from 0.3 to 2.5.

### Role of ABCB1 in neuroblastoma cell sensitivity to tozasertib and alisertib

Tozasertib was previously suggested to be an ABCB1 substrate [28], [29]. Indeed, all ABCB1-expressing cells among our cell line panel displayed substantially enhanced tozasertib IC_50_ values compared to the respective parental cell lines that do not express significant amounts of ABCB1: UKF-NB-2^r^DOX^20^, 7.6-fold; UKF-NB-2^r^VCR^10^, 9.6-fold, UKF-NB-3^r^DOX^20^, 92.2-fold, UKF-NB-3^r^VCR^10^, 77.7-fold; UKF-NB-6^r^VCR^10^, 72.9-fold (Table S1). In concordance, treatment with the ABCB1 inhibitor zosuquidar strongly sensitised these cell lines to tozasertib (Figure 1B, Table S2).

While the average tozasertib IC_50_ differed significantly between cells that show high ABCB1 expression and those that show low ABCB1 expression, there was no significant difference in the average alisertib IC_50_s (Figure 1C). In accordance, the tozasertib IC_50_ was increased in UKF-NB-3 cells transduced with a lentiviral vector encoding for ABCB1 (UKF-NB-3^ABCB1^) but the alisertib IC_50_ was not (Figure 1D). The ABCB1 inhibitor zosuquidar resensitised UKF-NB-3^ABCB1^ cells to tozasertib (Table S3). These data suggest that ABCB1 confers resistance to tozasertib but not to alisertib.

### Role of p53 in neuroblastoma cell sensitivity to tozasertib and alisertib

The role of p53 in the cancer cell response to aurora kinase inhibitors is not clear and may depend on the cellular context [10], [30]–[38].

**Figure 2:**
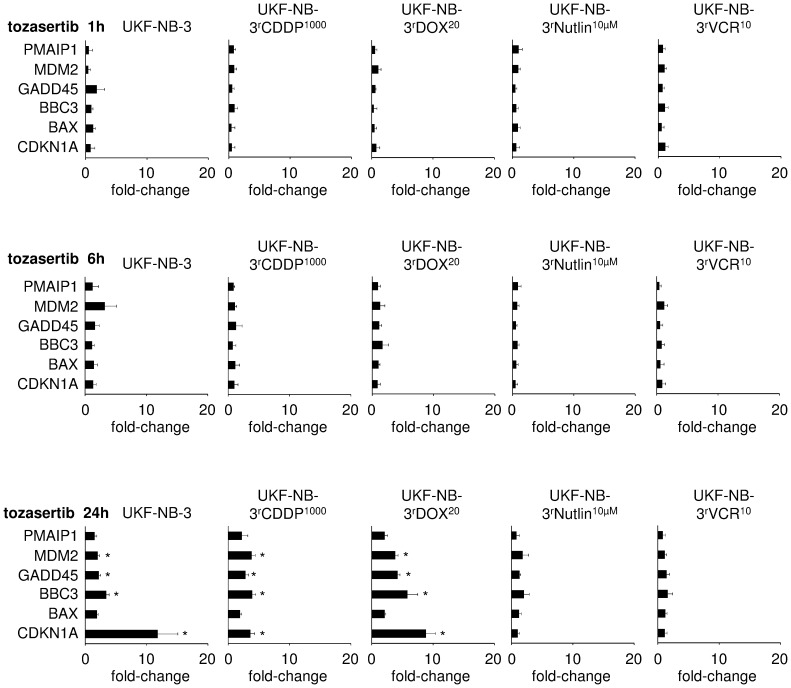
Tozasertib (1 µM)-induced expression of p53 target genes as indicated by qPCR. Expression levels are presented as fold change relative to non-treated controls. * P<0.05 relative to non-treated control.

**Figure 3:**
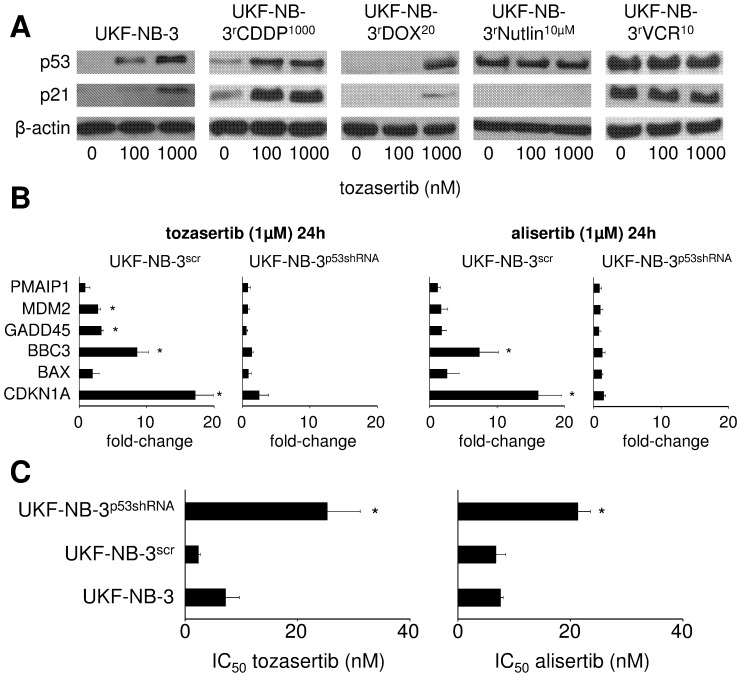
Role of p53 signalling in response to aurora kinase inhibition. A) tozasertib-induced p53 and p21 expression as indicated by Western blot after 24 h of incubation; B) tozasertib- and alisertib-induced expression of p53 target genes in UKF-NB-3 cells in which p53 was depleted using a lentiviral vector encoding shRNA directed against p53 (UKF-NB-3^p53shRNA^) or in UKF-NB-3 cells transduced with a control vector encoding non-targeting scramble shRNA (UKF-NB-3^scr^) as indicated by qPCR after 24 h. Expression levels are presented as fold change relative to non-treated controls. * P<0.05 relative to non-treated control; C) tozasertib and alisertib concentrations that reduce UKF-NB-3, UKF-NB-3^scr^, and UKF-NB-3^p53shRNA^ viability by 50% (IC_50_). * P<0.05 relative to UKF-NB-3 cells.

Tozasertib and alisertib induced a p53 response in the p53 wild-type cells UKF-NB-3, UKF-NB-3^r^CDDP^1000^, and UKF-NB-3^r^DOX^20^ but not in the p53-mutant cell lines UKF-NB-3^r^Nutlin^10µM^ and UKF-NB-3^r^VCR^10^ as indicated by qPCR. The expression of p53 target genes became detectable after 24 h of incubation with tozasertib (Figure 2) or alisertib (Figure S1). The p53 response was confirmed at the protein level by Western blot in tozasertib-treated UKF-NB-3, UKF-NB-3^r^CDDP^1000^, UKF-NB-3^r^DOX^20^, UKF-NB-3^r^Nutlin^10µM^, and UKF-NB-3^r^VCR^10^ (Figure 3A).

Some reports had suggested that the anti-cancer effects of aurora kinase inhibitors may depend on p73 activation in addition to p53 activation [39], [40] or that a p73 response may even replace a p53 response in p53-deficient cells [32]. In our system, however, RNAi-mediated depletion of p53 using a lentiviral vector encoding for shRNA directed against p53 abrogated the tozasertib- and alisertib-induced p53 response (Figure 3B) and reduced neuroblastoma sensitivity to tozasertib and alisertib (Figure 3C).

**Figure 4:**
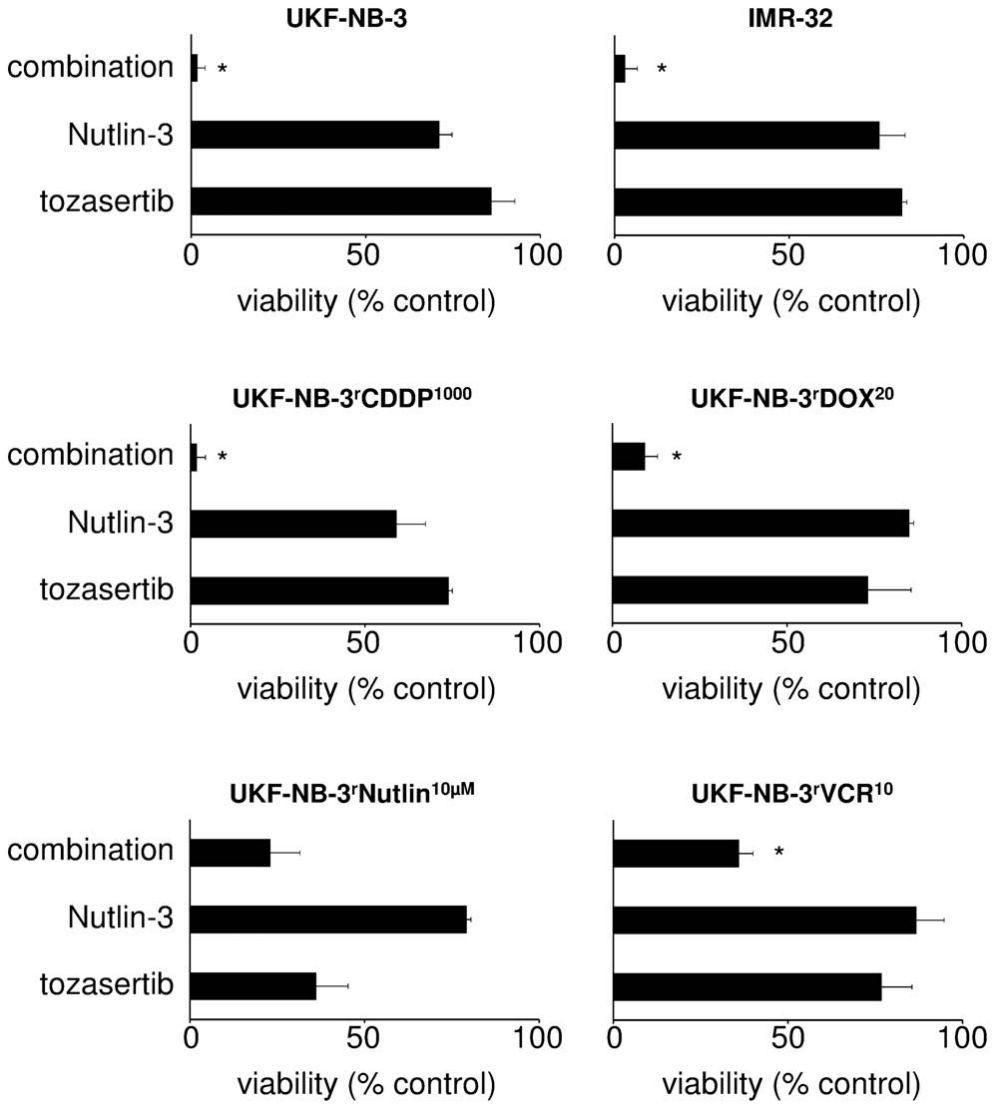
Effects of tozasertib on the viability of neuroblastoma cells in combination with the MDM2 inhibitor nutlin-3. Neuroblastoma cells were treated for five days with tozasertib, nutlin-3, or their combination. Cell viability was determined by MTT assay. The drug concentrations were: UKF-NB-3, tozasertib 6 nM, Nutlin-3 0.625 µM; IMR-32, tozasertib 6 nM, Nutlin-3 1.25 µM; UKF-NB-3^r^CDDP^1000^, tozasertib 6 nM, Nutlin-3 1.25 µM; UKF-NB-3^r^DOX^20^, tozasertib 156 nM, Nutlin-3 2.5 µM; UKF-NB-3^r^Nutlin^10µM^, tozasertib 156 nM, Nutlin-3 5 µM; UKF-NB-3^r^VCR^10^, tozasertib 156 nM, Nutlin-3 5 µM. * P<0.05 relative to either single treatment.

In order to further investigate the role of p53 in the effects of the aurora kinase inhibitors on the viability of the neuroblastoma cell lines, we combined tozasertib with the p53 activator nutlin-3 that interferes with the interaction of p53 and its endogenous inhibitor MDM2 through MDM2 binding [41]. Nutlin-3 significantly increased the tozasertib-induced effects in p53 wild-type UKF-NB-3 and UKF-NB-3^r^CDDP^1000^ cells (Figure 4). Similar results were obtained in p53 wild-type IMR-32 neuroblastoma cells (Figure 4). However, nutlin-3 did not enhance tozasertib activity in p53-mutated UKF-NB-3^r^Nutlin^10µM^ cells (Figure 4). Nutlin-3 also enhanced the effects of tozasertib in p53-mutated UKF-NB-3^r^VCR^10^ cells (Figure 4). This is most probably due to interaction of nutlin-3 with the ABCB1-mediated tozasertib efflux in the highly ABCB1-expressing UKF-NB-3^r^VCR^10^ cells. Nutlin-3 is known to interfere with ABCB1-mediated drug transport [42] and the ABCB1 inhibitor zosuquidar induced similar effects (Figure S2).

### Effects of tozasertib on aurora kinase function, the neuroblastoma cell cycle, and neuroblastoma cell apoptosis

**Figure 5:**
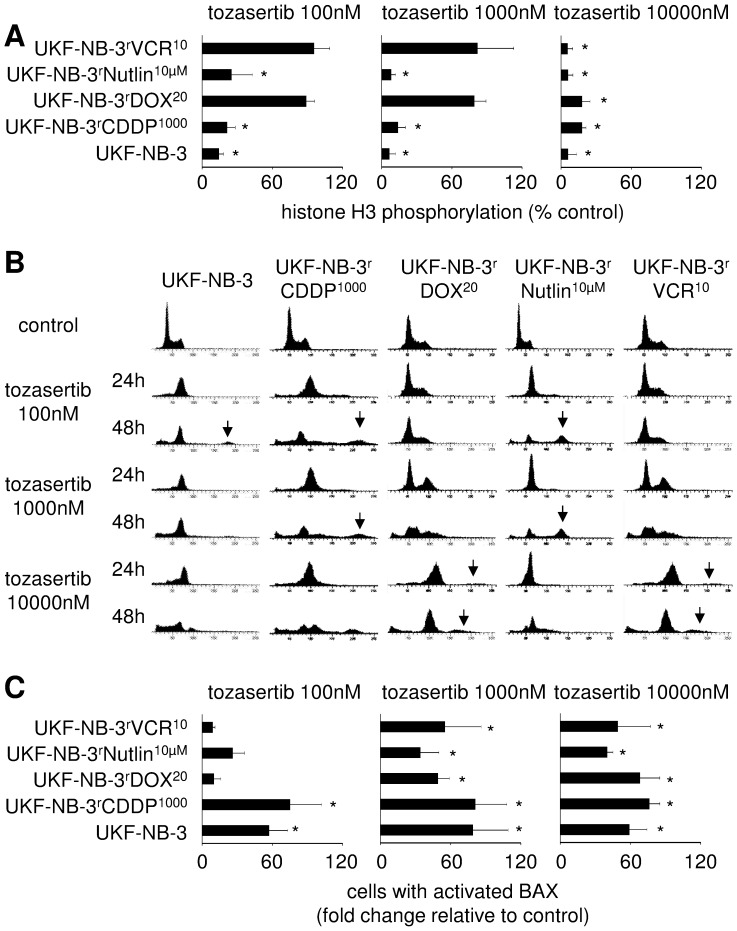
Effects of tozasertib on histone H3 phosphorylation (indicating aurora kinase A and B activity), cell cycle distribution, and apoptosis (indicated by BAX activation) in neuroblastoma cells. A) Numbers of cells expressing phosphorylated histone H3 were determined flow cytometry; * P<0.05 relative to untreated control; B) Representative histograms indicating cell cycle distribution in neuroblastoma cells after tozasertib treatment, arrows indicate additional peaks with high DNA content, possibly indicating endoreduplication; C) Numbers of cells with activated BAX expressed as fold change relative to control as determined by flow cytometry using an antibody specific for activated BAX after 24 h of tozasertib treatment. * P<0.05 relative to untreated control.

Histon H3 is phosphorylated by aurora kinases A and B. Therefore, determination of histon H3 phosphorylation can serve as a surrogate for determining effects on aurora kinase function [17], [43]. Substantially higher tozasertib concentrations were needed to suppress histone H3 phosphorylation in the ABCB1-expressing cell lines UKF-NB-3^r^DOX^20^ and UKF-NB-3^r^VCR^10^ than in the low ABCB1-expressing cell lines UKF-NB-3, UKF-NB-3^r^CDDP^1000^, and UKF-NB-3^r^Nutlin^10µM^ (Figure 5A). This finding is in concert with the action of ABCB1 as efflux transporter that limits the intracellular tozasertib levels. The fact that histone H3 phosphorylation similarly affected p53 wild-type UKF-NB-3 and UKF-NB-3^r^CDDP^1000^ cells and p53-mutant UKF-NB-3^r^Nutlin^10µM^ suggests that p53 inactivation reduces tozasertib sensitivity by interfering with the drug-induced signalling downstream of aurora kinase inhibition. In accordance, the effects of tozasertib on histone H3 phosphorylation were similar among the ABCB1-expressing cell lines UKF-NB-3^r^DOX^20^ and UKF-NB-3^r^VCR^10^ (Figure 5A).

Tozasertib induced a G2/M cell cycle block in p53 wild-type and p53-mutant neuroblastoma cell lines but with different kinetics and concentration-dependencies (Figure 5B) and induced more or less pronounced signs of endoreduplication (Figure 5B).

**Figure 6:**
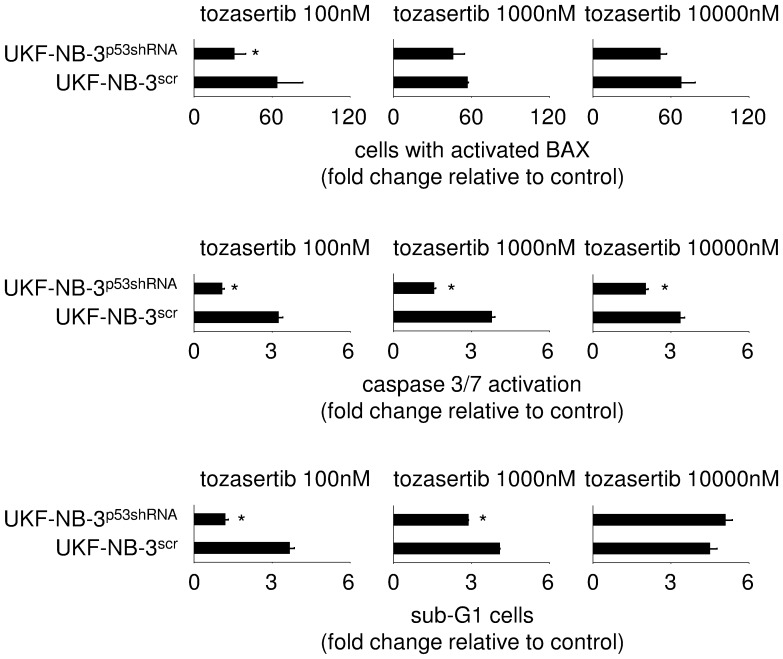
Tozasertib-induced apoptosis in UKF-NB-3 cells transduced with a lentiviral vector encoding shRNA directed against p53 (UKF-NB-3^p53shRNA^) and UKF-NB-3 transduced with a control vector expressing non-targeting scrambled shRNA (UKF-NB-3^scr^) as indicated by BAX activation, caspase 3/7 activation, and determination of sub-G1 cells. * P<0.05 relative to UKF-NB-3^scr^.

Determination of BAX activation suggested that tozasertib induces apoptosis in all cell lines (Figure 5C). Higher tozasertib concentrations were necessary to induce BAX activation in ABCB1-expressing UKF-NB-3^r^DOX^20^, p53-mutant UKF-NB-3^r^Nutlin^10µM^ cells, and ABCB1-expressing and p53-mutated UKF-NB-3^r^VCR^10^ cells than in low ABCB1-expressing and p53 wild-type UKF-NB-3 and UKF-NB-3^r^CDDP^1000^ cells. In accordance, p53-depleted UKF-NB-3^p53shRNA^ cells displayed lower BAX activation than control vector-transduced UKF-NB-3^scr^ cells (Figure 6A). Similar results were obtained by the detection of caspase 3/7 activity (Figure 6B) and the fraction of sub-G1 cells (Figure 6B).

Taken together, these data suggest that the action of aurora kinase inhibitors is at least in part mediated through cell cycle inhibition and apoptosis induction as consequence of aurora kinase inhibition.

## Discussion

Testing of tozasertib, a pan aurora kinase inhibitor [16], and alisertib, a second generation aurora kinase inhibitor that inhibits aurora kinase A and B with a higher affinity to aurora kinase A [17], in a panel of drug-resistant neuroblastoma cell lines revealed differing activity profiles. ABCB1 expression reduced cancer cell sensibility to tozasertib but not to alisertib. This is in concert with previous findings that had suggested tozasertib to be a substrate of ABCB1 [28], [29]. Moreover, we provided evidence that alisertib is not transported by ABCB1. The anti-neuroblastoma mechanism included (at least in part) aurora kinase inhibition as indicated by reduced phosporylation of the aurora kinase substrate histone H3, cell cycle inhibition, and induction of apoptosis.

Varying findings have been published on the involvement of p53 in the aurora kinase inhibitor-induced anti-cancer effects in models from various cancer entities. Various reports showed that aurora kinase inhibitors activate p53 signalling and that this p53 signalling contributed to the aurora kinase inhibitor-induced anti-cancer effects [10], [33]–[35]. Other reports suggested that p53 may be of minor relevance for aurora kinase inhibitor activity [32], [36], [37] or that aurora kinase inhibitor activity may be enhanced in p53-defective cells [30], [31], [38]. Also, the role of p53 may differ between approaches that target aurora kinase A and those that target aurora kinase B [44]. Thus, the relevance of p53 in response to aurora kinase inhibition apparently depends on the cellular context.

In neuroblastoma cells, the aurora kinase A and B inhibitor CCT137690 was described to induce a p53 response [10]. Our results obtained in p53 wild-type and p53-mutant cells as well as in p53-depleted cells indicated that p53 activation is of relevance for the anti-cancer effects exerted by aurora kinase inhibitors in neuroblastoma cells. The combination of the MDM2 inhibitor and p53 activator nutlin-3 with tozasertib enhanced the activity of aurora kinase inhibitors in in the presence of functional p53. This is of clinical relevance since p53 mutations were described as acquired resistance mechanism in neuroblastoma [45], [46]. Nevertheless, the vast majority of neuroblastomas (about 85%) harbours p53 wild-type cells [45], [46].

Nutlin-3 also enhanced the tozasertib-induced effects in p53-mutated ABCB1-expressing UKF-NB-3^r^VCR^10^ cells. Since nutlin-3 interferes with ABCB1-mediated drug efflux [42] this is most probably due to nutlin-3-mediated inhibition of ABCB1-mediated tozasertib efflux. Therefore, nutlin-3 may enhance tozasertib efficacy through p53 activation and inhibition of ABCB1-mediated tozasertib efflux.

Noteworthy, the combined effects of aurora kinase inhibitors and MDM2 inhibitors may depend on the sequence of drug administration. Previous investigations in p53 wild-type A375 melanoma cells had revealed that nutlin-3 pre-treatment had resulted in a p53-mediated cell cycle arrest that protected these cells from tozasertib-induced anti-cancer effects while tozasertib pretreatment or simultaneous combined tozasertib and nutlin-3 treatment had resulted in enhanced combined anti-cancer effects [39]. Nutlin-3 pre-treatment had also protected A549 lung cancer cells, primary human keratinocytes, and HCT116p53+/+ colorectal cancer cells (but not HCT116p53−/− cells) from tozasertib-induced toxicity [39]. In this context, we investigated the effects of simultaneous tozasertib and nutlin-3 treatment in primary human foreskin fibroblasts (Figure S2). The results were promising because 1) the primary fibroblasts were much less sensitive to tozasertib and nutlin-3 than p53 wild-type and p53-mutant neuroblastoma cells and 2) the combination of tozasertib and nutlin-3 resulted in contrast to the effects observed in p53 wild-type neuroblastoma cells not in enhanced toxicity compared to either single treatment (Figure S2).

Finally, it needs to be noted that although the major body of data from our study clearly demonstrated that p53 function was critically involved in the neuroblastoma cell response to aurora kinase inhibition, the p53-mutated cell line UKF-NB-6^r^Nutlin^10µM^ was similarly sensitive to tozasertib and alisertib as the p53 wild-type neuroblastoma cell lines. The reasons for this remain unclear and emphasise that many factors may determine neuroblastoma cell sensitivity to aurora kinase inhibitors in addition to the p53 status. Possibly, aurora kinase inhibitor-induced p73 activation [32] and/or other events that need to be determined in future studies may be responsible for this. Noteworthy, kinase inhibitors may interfere with other (previously unidentified) kinases in addition to the target kinases they were designed to inhibit. For example, tozasertib was shown to interfere with additional kinases including ABL and FLT3 [16], [47], [48]. Although the similarity of the effects exerted by two structurally different aurora kinase inhibitors suggests aurora kinases to be relevant common targets, effects on other kinases may contribute to the effects of tozasertib and/or alisertib on the viability of (certain) neuroblastoma cells.

Taken together, we provide the first data on the efficacy of aurora kinase inhibitors in neuroblastoma cells with acquired resistance to anti-cancer drugs. Our data suggest that aurora kinases represent a therapeutic target in therapy-refractory neuroblastoma cells, in particular in p53 wild-type therapy-refractory neuroblastoma cells.

## Supporting Information

Figure S1 **Alisertib-induced expression of p53 target genes in parental UKF-NB-3 cells and their drug-resistant sub-lines as indicated by qPCR.**
(PDF)

Figure S2 **Effects of tozasertib combination therapies on the viability of UKF-NB-3^r^VCR^10^ cells (tozasertib plus the ABCB1 inhibitor zosuquidar) or primary human foreskin fibroblasts (HFFs, tozasertib plus the MDM2 inhibitor nutlin-3) as determined by MTT assay after 5 days of incubation.**
(PDF)

Table S1 **Concentrations of tozasertib and alisertib that decrease neuroblastoma cell viability by 50% (IC_50_).**
(PDF)

Table S2 **Concentrations of tozasertib that decrease the viability of neuroblastoma cells by 50% (IC_50_) in the presence of the ABCB1 inhibitor zosuquidar.**
(PDF)

Table S3 **Concentrations of tozasertib that decrease the viability of UKF-NB-3 cells, ABCB1-transduced UKF-NB-3 cells, or control vector-transduced UKF-NB-3 cells by 50% (IC_50_) in the presence of the ABCB1 inhibitor zosuquidar.**
(PDF)
