# Supplementary material for: Aurora Kinases as Targets in Drug-Resistant Neuroblastoma Cells
Source: PLoS One. 2014 Sep 30;9(9):e108758. doi: 10.1371/journal.pone.0108758 (PMC4182628; doi:10.1371/journal.pone.0108758)
Supplement: Figure S1 — Alisertib-induced expression of p53 target genes in parental UKF-NB-3 cells and their drug-resistant sub-lines as indicated by qPCR. (PDF) [file pone.0108758.s001.pdf]

Figure S1

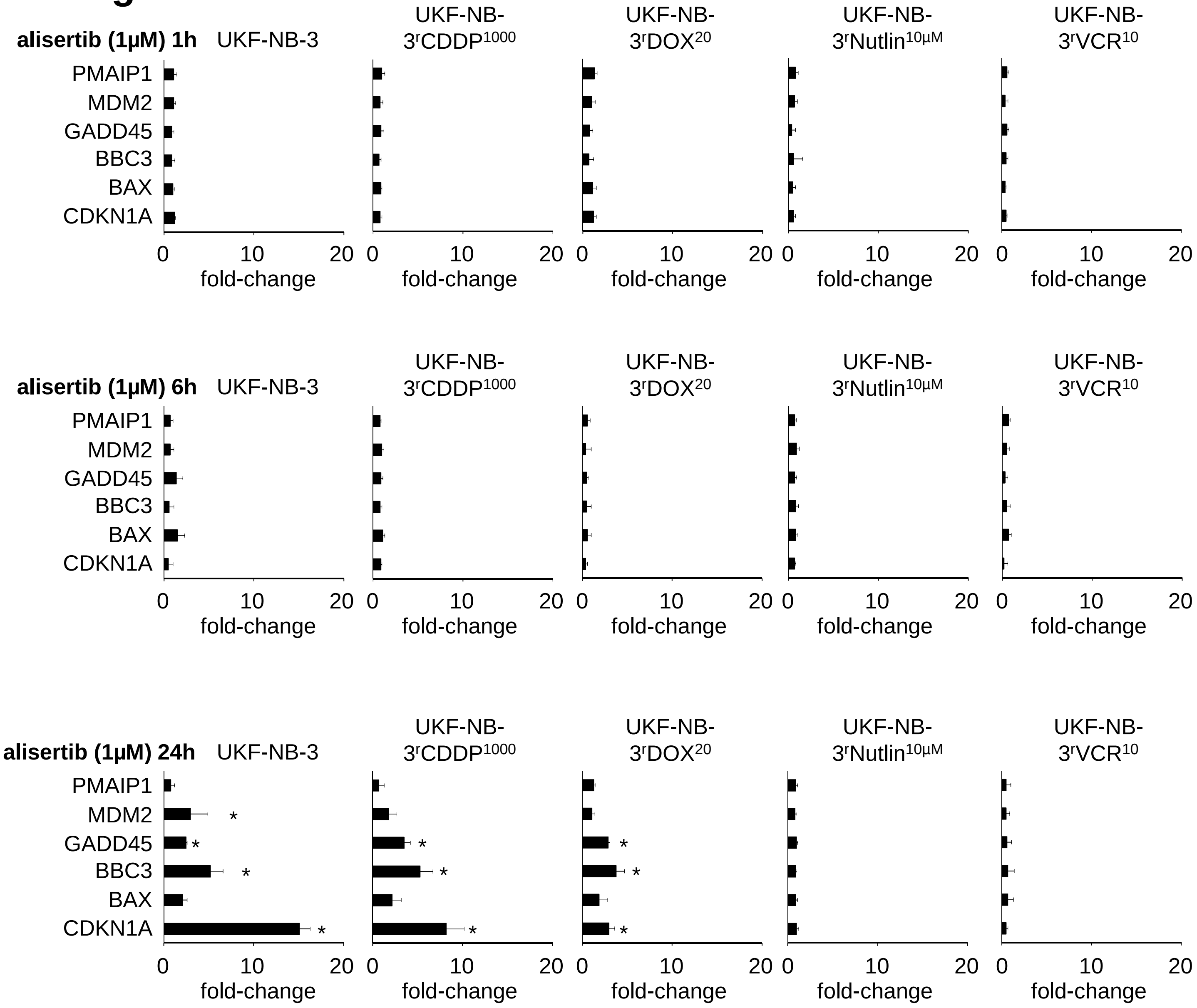
alisertib (1μM) 1h
alisertib (1μM) 6h
alisertib (1μM) 24h
UKF-NB-3
UKF-NB-3^{r}CDDP^{1000}
UKF-NB-3^{r}DOX^{20}
UKF-NB-3^{r}Nutlin^{10μM}
UKF-NB-3^{r}VCR^{10}
PMAIP1
MDM2
GADD45
BBC3
BAX
CDKN1A
0
10
20
fold-change
*

Figure S1. Alisertib-induced expression of p53 target genes as indicated by qPCR. Expression levels are presented as fold change relative to non-treated controls. * $P < 0.05$ relative to non-treated control
