# Supplementary material for: Aurora Kinases as Targets in Drug-Resistant Neuroblastoma Cells
Source: PLoS One. 2014 Sep 30;9(9):e108758. doi: 10.1371/journal.pone.0108758 (PMC4182628; doi:10.1371/journal.pone.0108758)
Supplement: Figure S2 — Effects of tozasertib combination therapies on the viability of UKF-NB-3rVCR10 cells (tozasertib plus the ABCB1 inhibitor zosuquidar) or primary human foreskin fibroblasts (HFFs, tozasertib plus the MDM2 inhibitor nutlin-3) as determined by MTT assay after 5 days of incubation. (PDF) [file pone.0108758.s002.pdf]

Figure S2

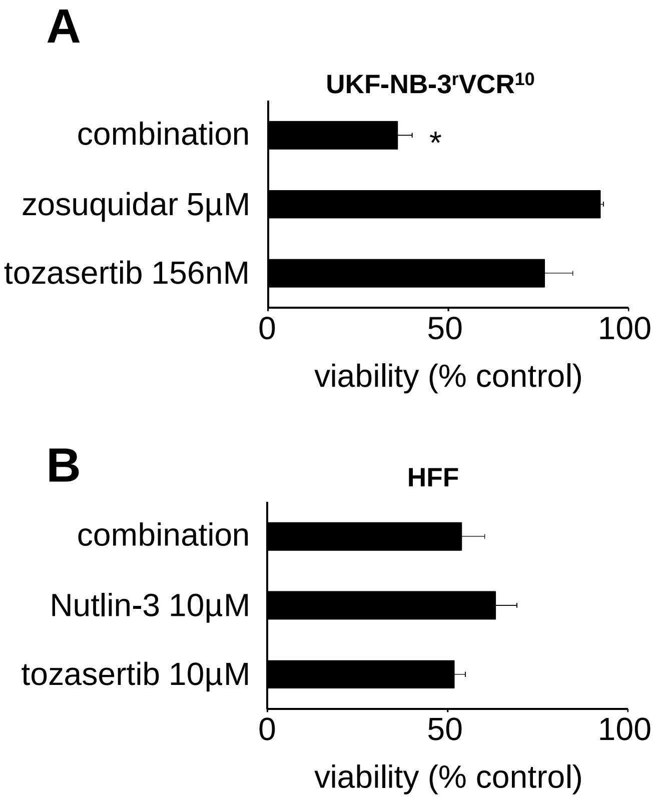

Figure S2. Effects of tozasertib combination therapies on cell viability as determined by MTT assay after 5 days of incubation. A) ABCB1-expressing UKF-NB-3^{r}VCR^{10} were treated with tozasertib, the ABCB1 inhibitor zosuquidar, or their combination. B) Primary human foreskin fibroblasts were treated with tozasertib, the MDM2 inhibitor nutlin-3, or their combination. * $P < 0.05$ relative to either single treatment.
