# Supplementary material for: Aurora Kinases as Targets in Drug-Resistant Neuroblastoma Cells
Source: PLoS One. 2014 Sep 30;9(9):e108758. doi: 10.1371/journal.pone.0108758 (PMC4182628; doi:10.1371/journal.pone.0108758)
Supplement: Table S1 — Concentrations of tozasertib and alisertib that decrease neuroblastoma cell viability by 50% (IC50). (PDF) [file pone.0108758.s003.pdf]

Table S1. Concentrations of tozasertib and alisertib that decrease neuroblastoma cell viability by 50% (IC$_{50}$) as indicated by MTT assay after 120h of incubation.

Cell line	ABCB1 expression	p53 status	IC$_{50}$ tozasertib (nM)	IC$_{50}$ alisertib (nM)
UKF-NB-2	low	wild-type	73.4 ± 18.2	20.8 ± 1.2
UKF-NB-3	low	wild-type	7.2 ± 2.4	7.6 ± 0.5
UKF-NB-6	low	wild-type	5.5 ± 0.4	12.4 ± 0.2
UKF-NB-3^{r}CDDP^{1000}	low	wild-type	10.8 ± 2.9 (1.5)[1]	2.0 ± 0.7 (0.3)
UKF-NB-6^{r}CDDP^{2000}	low	wild-type	60.8 ± 1.2 (11.1)	24.9 ± 3.1 (2.0)
UKF-NB-2^{r}DOX^{20}	high	wild-type	558.2 ± 52.7 (7.6)	26.8 ± 1.3 (1.3)
UKF-NB-3^{r}DOX^{20}	high	wild-type	664.0 ± 257.8 (92.2)	8.9 ± 2.0 (1.2)
UKF-NB-2^{r}Nutlin$^{10\mu M}$	low	wild-type	220.4 ± 30.8 (3.0)	41.5 ± 2.7 (2.0)
UKF-NB-3^{r}Nutlin$^{10\mu M}$	low	G245C mutation	24.3 ± 4.0 (3.4)	15.7 ± 3.2 (2.1)
UKF-NB-6^{r}Nutlin$^{10\mu M}$	low	K132N, P223L mutation	7.6 ± 0.2 (1.4)	11.3 ± 1.0 (0.9)
UKF-NB-2^{r}VCR^{10}	high	wild-type	702.9 ± 85.1 (9.6)	27.5 ± 5.7 (1.3)
UKF-NB-3^{r}VCR^{10}	high	C135F mutation	559.3 ± 130.8 (77.7)	19.3 ± 3.4 (2.5)
UKF-NB-6^{r}VCR^{10}	high	wild-type	400.8 ± 90.4 (72.9)	22.1 ± 0.6 (1.8)

[1] relative resistance compared to respective parental cell line (IC$_{50}$ resistant sub-line/ IC$_{50}$ respective parental cell line)
