# Supplementary material for: Aurora Kinases as Targets in Drug-Resistant Neuroblastoma Cells
Source: PLoS One. 2014 Sep 30;9(9):e108758. doi: 10.1371/journal.pone.0108758 (PMC4182628; doi:10.1371/journal.pone.0108758)
Supplement: Table S2 — Concentrations of tozasertib that decrease the viability of neuroblastoma cells by 50% (IC50) in the presence of the ABCB1 inhibitor zosuquidar. (PDF) [file pone.0108758.s004.pdf]

Table S2. Concentrations of tozasertib that decrease neuroblastoma cell viability by 50% (IC_{50}) as indicated by MTT assay after 120h of incubation in the presence of the ABCB1 inhibitor zosuquidar (5µM)[1].

	**tozasertib alone**	**tozasertib plus zosuquidar**
**Cell line**	**IC_{50} tozasertib (nM)**	**IC_{50} tozasertib (nM)**
UKF-NB-2	73.4 ± 18.2	27.0 ± 1.9 [2.7]
UKF-NB-3	7.2 ± 2.4	6.5 ± 2.6 [1.1]
UKF-NB-6	5.5 ± 0.4	2.9 ± 0.2 [1.9]
UKF-NB-3^{r}CDDP^{1000}	10.8 ± 2.9 (1.5)[2]	9.9 ± 3.9 (1.5) [1.1][3]
UKF-NB-6^{r}CDDP^{2000}	60.8 ± 1.2 (11.1)	33.5 ± 0.1 (11.5) [1.8]
UKF-NB-2^{r}DOX^{20}	558.2 ± 52.7 (7.6)	124.3 ± 6.0 (4.6) [4.5]
UKF-NB-3^{r}DOX^{20}	664.0 ± 257.8 (92.2)	26.2 ± 8.8 (4.0) [25.3]
UKF-NB-2^{r}Nutlin^{10µM}	220.4 ± 30.8 (3.0)	150.5 ± 63.8 (5.6) [1.5]
UKF-NB-3^{r}Nutlin^{10µM}	24.3 ± 4.0 (3.4)	23.6 ± 2.8 (3.6) [1.0]
UKF-NB-6^{r}Nutlin^{10µM}	7.6 ± 0.2 (1.4)	5.2 ± 0.4 (1.8) [1.5]
UKF-NB-2^{r}VCR^{10}	702.9 ± 85.1 (9.6)	21.6 ± 0.4 (0.8) [32.5]
UKF-NB-3^{r}VCR^{10}	559.3 ± 130.8 (77.7)	38.9 ± 6.6 (6.0) [14.4]
UKF-NB-6^{r}VCR^{10}	400.8 ± 90.4 (72.9)	50.9 ± 7.0 (17.6) [7.9]

[1] Zosuquidar 5µM alone did not exert significant effects on neuroblastoma cell viability.
[2] relative resistance compared to respective parental cell line (IC_{50} resistant sub-line/ IC_{50} respective parental cell line)
[3] fold sensitisation by zosuquidar (IC_{50} without zosuquidar/ IC_{50} with zosuquidar]
