# Supplementary material for: Aurora Kinases as Targets in Drug-Resistant Neuroblastoma Cells
Source: PLoS One. 2014 Sep 30;9(9):e108758. doi: 10.1371/journal.pone.0108758 (PMC4182628; doi:10.1371/journal.pone.0108758)
Supplement: Table S3 — Concentrations of tozasertib that decrease the viability of UKF-NB-3 cells, ABCB1-transduced UKF-NB-3 cells, or control vector-transduced UKF-NB-3 cells by 50% (IC50) in the presence of the ABCB1 inhibitor zosuquidar. (PDF) [file pone.0108758.s005.pdf]

Table S3. Concentrations of tozasertib that decrease neuroblastoma cell viability by 50% (IC_{50}) as indicated by MTT assay after 120h of incubation in the presence of the ABCB1 inhibitor zosuquidar (5µM)[1].

	**tozasertib alone**	**tozasertib plus zosuquidar**
**Cell line**	**IC_{50} tozasertib (nM)**	**IC_{50} tozasertib (nM)**
UKF-NB-3	7.2 ± 2.4	7.6 ± 0.5 [0.9][2]
UKF-NB-3^{control}	5.6 ± 0.4 (0.8)[3]	10.4 ± 1.6 (1.4) [1.9]
UKF-NB-3^{ABCB1}	395.8 ± 46.0 (55.0)	10.0 ± 0.5.8 (1.3) [39.5]

[1] Zosuquidar 5µM alone did not exert significant effects on neuroblastoma cell viability.
[2] fold sensitisation by zosuquidar (IC_{50} without zosuquidar/ IC_{50} with zosuquidar]
[3] relative resistance compared to respective parental cell line (IC_{50} resistant sub-line/ IC_{50} respective parental cell line)
